# Phenotypic and Genotypic Features of *Klebsiella pneumoniae* Harboring Carbapenemases in Egypt: OXA-48-Like Carbapenemases as an Investigated Model

**DOI:** 10.3390/antibiotics9120852

**Published:** 2020-11-28

**Authors:** Suzan Mohammed Ragheb, Mahmoud Mohamed Tawfick, Amani Ali El-Kholy, Abeer Khairy Abdulall

**Affiliations:** 1 Department of Microbiology and Immunology, Faculty of Pharmacy, Modern University for Technology and Information (MTI), Cairo 11571, Egypt; drsuzanragheb84@gmail.com; 2 Department of Microbiology and Immunology, Faculty of Pharmacy (Boys), Al-Azhar University, Cairo 11884, Egypt; 3 Department of Microbiology, Faculty of Pharmacy, Heliopolis University, Cairo 11785, Egypt; 4 Department of Clinical and Chemical Pathology, Faculty of Medicine, Cairo University, Cairo 11562, Egypt; aaakholy@gmail.com; 5 Department of Microbiology and Immunology, Faculty of Pharmacy (Girls), Al-Azhar University, Cairo 11884, Egypt

**Keywords:** *Klebsiella pneumoniae*, carbapenemases, *bla*_OXA-48_, ERIC-PCR, plasmid profile analysis, biofilm formation

## Abstract

This study aimed at the characterization of carbapenem-resistant *Klebsiella pneumoniae* isolates focusing on typing of the *bla*_OXA-48-like_ genes. Additionally, the correlation between the resistance pattern and biofilm formation capacity of the carbapenem-resistant *K. pneumoniae* isolates was studied. The collected isolates were assessed for their antimicrobial resistance and carbapenemases production by a modified Hodge test and inhibitor-based tests. The carbapenemases encoding genes (*bla*_KPC_, *bla*_NDM_, *bla*_VIM_, *bla*_IMP_, and *bla*_OXA-48-like_) were detected by PCR. Isolates harboring *bla*_OXA-48-like_ genes were genotyped by Enterobacterial Repetitive Intergenic Consensus-Polymerase Chain Reaction (ERIC-PCR) and plasmid profile analysis. The discriminatory power of the three typing methods (antibiogram, ERIC-PCR, and plasmid profile analysis) was compared by calculation of Simpson’s Diversity Index (SDI). The transferability of *bla*_OXA-48_ gene was tested by chemical transformation. The biofilm formation capacity and the prevalence of the genes encoding the fimbrial adhesins *(fimH-1* and *mrkD*) were investigated. The isolates showed remarkable resistance to β-lactams and non-β-lactams antimicrobials. The coexistence of the investigated carbapenemases encoding genes was prevalent except for only 15 isolates. The plasmid profile analysis had the highest discriminatory power (SDI = 0.98) in comparison with ERIC-PCR (SDI = 0.89) and antibiogram (SDI = 0.78). The transferability of *bla*_OXA-48_ gene was unsuccessful. All isolates were biofilm formers with the absence of a significant correlation between the biofilm formation capacity and resistance profile. The genes *fimH-1* and *mrkD* were prevalent among the isolates. The prevalence of carbapenemases encoding genes, especially *bla*_OXA-48-like_ genes in Egyptian healthcare settings, is worrisome and necessitates further strict dissemination control measures.

## 1. Introduction

Antimicrobial resistance is among the serious problems that contribute to high morbidity and mortality rates, particularly in immunocompromised patients [1]. Several alarming reports released by international organizations have described the consequences of this crisis at health and economic levels [2,3,4]. *Klebsiella pneumoniae* is among the ESKAPE pathogens (*Enterococcus faecium*, *Staphylococcus aureus*, *Klebsiella pneumoniae*, *Acinetobacter baumannii*, *Pseudomonas aeruginosa*, and *Enterobacter* spp.) which adopt numerous mechanisms to “escape” from different antimicrobials actions [5]. Increased attention has emerged toward resistance to carbapenems, which are considered as the last resort β-lactam for treatment of life-threatening infections caused by multidrug-resistant Enterobacteriaceae. Thus, the WHO Global priority list of antibiotic-resistant bacteria classified carbapenem-resistant Enterobacteriaceae as pathogens of critical priority [6].

Carbapenem resistance may be attributed to porin mutations, efflux pumps, and/or carbapenemases production. Although there is an expansion in the number of emerged carbapenemases, five important members that belong to three Ambler classes are the most studied, namely, *Klebsiella pneumoniae* carbapenemase*s (bla*_KPC_), New Delhi metallo-β-lactamases (*bla*_NDM_), Verona integron-encoded metallo-β-lactamase*s* (*bla*_VIM_), Active on imipenem metallo-β-lactamases (*bla*_IMP_), and Oxacillinase-48-like carbapenemases (*bla*_OXA-48-like_) [7,8]. These carbapenemases have several variants according to the National Center for Biotechnology Information (NCBI) Antimicrobial Resistance Reference Gene database, available at https://www.ncbi.nlm.nih.gov/pathogens/isolates#/refgene. Additionally, and in the terms of the geographical dissemination, the ”big five” carbapenemases differ in their spread globally, and their epidemiological status may be endemic, or just recorded cases [8]. KPC β-lactamases belong to the Ambler Class A carbapenemases which have serine at their active site. In addition to its activity against carbapenems, *bla*_KPC_ show activity against a wide range of β-lactams as penicillins, cephalosporins, and monobactams, and also overcome the in-vitro activity of β-lactamase inhibitors such as clavulanic acid and sulbactam. Additionally, plasmids carrying *bla*_KPC_ encoding gene may carry other genes that confer resistance to other antimicrobial classes such as fluoroquinolones and aminoglycosides [9]. NDM, VIM, and IMP metallo β-lactamases are the most common metallo-β-lactamases that belong to the Ambler Class B carbapenemases with zinc at their active site, and this leads to their inhibition by metal-chelating agents such as ethylenediaminetetraacetic acid (EDTA). They show resistance to penicillins, cephalosporins, and carbapenems and also to β-lactamase inhibitors. Furthermore, these carbapenemases are supported by genetic platforms that allow their dissemination with other resistance determinants of fluoroquinolones and aminoglycosides [10]. OXA-48-like carbapenemases belong to the Ambler Class D carbapenemases with serine at their active site. They are not inhibited by EDTA or clavulanic acid, and this makes their detection challenging [11]. 

In Egypt, *bla*_OXA-48_ is of a great concern due to its endemicity in healthcare settings, especially in a successful candidate such as *K. pneumoniae*, which is characterized by a great plasmid load and high ability of transfer of resistance determinants of different antimicrobial classes [12]. There are several features concerning the *bla*_OXA-48_ gene. It is located on pOXA-48a IncL plasmid which does not carry any further antimicrobial determinants other than the *bla*_OXA-48_ gene. The *bla*_OXA-48_ gene is surrounded by two copies of the insertion sequence, IS*1999* [13]. Additionally, the rapid dissemination of pOXA-48a may be attributed to the disruption of *tir* gene (encodes a protein that is responsible for transfer inhibition) by transposon Tn*1999* and its variants [14]. Furthermore, *bla*_OXA-48_ gene has several variants that differ by few amino acids, and consequently differ in their hydrolysis specificities [13]. 

The dissemination of resistance genes, especially those encoding carbapenemases, necessitates the application of typing methods that participate in the study of the features and clonality of isolates that harbor various antimicrobials determinants [15].

Another problematic feature associated with carbapenem resistance in *K. pneumoniae* is biofilm formation. The International Union of Pure and Applied Chemistry defines biofilm as “an aggregate of microorganisms in which cells that are frequently embedded within a self-produced matrix of extracellular polymeric substance (EPS) adhere to each other and/or to a surface” [16]. Biofilm formation participates in the aggravation of serious clinical manifestations and leads to reduced susceptibility to antimicrobial agents [17]. Type 1 and 3 fimbriae have a crucial role in adhesion, which is a key factor in *K. pneumoniae* biofilm formation besides capsule and lipopolysaccharide [18].

In this study, we aimed to characterize carbapenem-resistant *K. pneumoniae* isolated from patients admitted to an Egyptian tertiary hospital. Additionally, we focused on the typing of the detected *bla*_OXA-48-like_ genes. Additionally, we shed light on the biofilm formation capacity of the carbapenem-resistant isolates and studied the possible correlation between the two traits

## 2. Results

### 2.1. Distribution and Identification of K. pneumoniae Isolates

**Figure 1:**
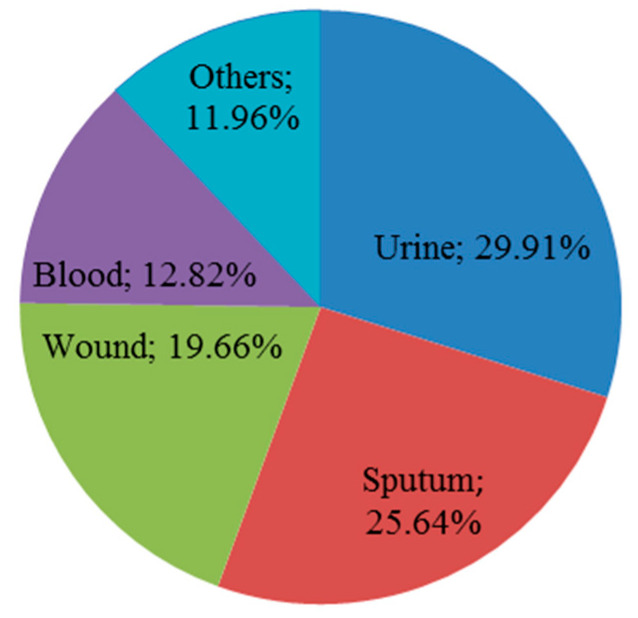
The distribution of collected isolates according to their origin: urine (29.91%), sputum (25.64%), wound (19.66%), blood (12.82%), and others (endotracheal aspirate (4.27%), central venous pressure tip (1.71%), cerebrospinal fluid (0.85%), pleural fluid (0.85%), drain culture (0.85%), bedsore (0.85%), bile culture (0.85%), central venous line culture (0.85%), and aspirate fluid (0.85%)).

A total of 117 isolates of carbapenem-non-susceptible *K. pneumoniae* were collected and recovered from diverse clinical specimens as shown in Figure 1. The confirmation of isolate identification using MALDI-TOF/MS had a high confidence ranging from 99.7 to 99.9%.

### 2.2. Isolate Antimicrobial Susceptibility Pattern

**Figure 2:**
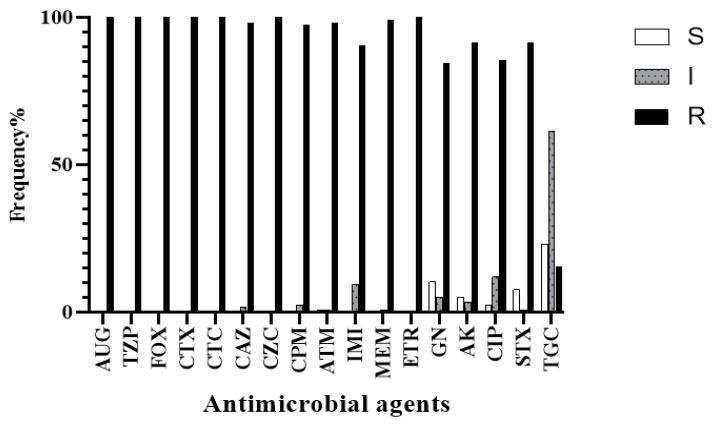
The frequencies of antimicrobial resistance of collected isolates to different antimicrobial classes. The frequencies of *K. pneumoniae* isolates resistance against β-lactams were very high (mostly 100%), while they were variable for non-β-lactam antimicrobials. S, Sensitive; I, Intermediate; R, Resistant. AUG, amoxicillin/clavulanic acid; TZP, piperacillin/tazobactam; FOX, cefoxitin; CTX, cefotaxime; CTC, cefotaxime/clavulanic acid; CAZ, ceftazidime; CZC, ceftazidime/clavulanic acid; CPM, cefepime; ATM, aztreonam; IMI, imipenem; MEM, meropenem; ETR, ertapenem; GN, gentamicin; AK, amikacin; CIP, ciprofloxacin; STX, sulfamethoxazole/trimethoprim, and (TGC) tigecycline. The interpretation of the results was according to the Clinical and Laboratory Standards Institute guidelines, CLSI (M100-S26, 2016). Interpretation of tigecycline results was according to the FDA recommendations available at http://www.accessdata.fda.gov/drugsatfda_docs/label/2009/021821s016lbl.pdf.

**Figure 3:**
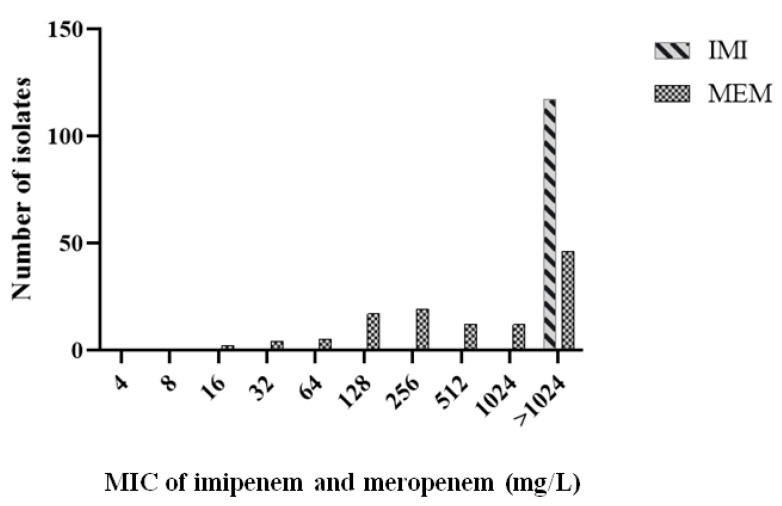
The distribution of the minimum inhibitory concentration MIC values of imipenem (IMI) and meropenem (MEM) among isolates. For both antimicrobial agents, the MICs for the resistant isolates were ≥4 mg/L according to CLSI (M100-S26, 2016) guidelines.

According to interim standard definitions of acquired resistance by Magiorakos et al. [19], our isolates were considered as multidrug-resistant (MDR) with the possibility of being extensively drug-resistant (XDR). As shown in Table S1, there was remarkable resistance to most antimicrobial agents. All isolates showed a resistant or intermediate profile against most β-Lactams such as amoxicillin/clavulanic acid (AUG), piperacillin/tazobactam (TZP), cefoxitin (FOX), cefotaxime (CTX), cefotaxime/clavulanic acid (CTC), ceftazidime (CAZ), ceftazidime/clavulanic acid (CZC), cefepime (CPM), imipenem (IMI), meropenem (MEM), and ertapenem (ETR). The susceptibilities of isolates to aztreonam (ATM), gentamicin (GN), amikacin (AK), ciprofloxacin (CIP), sulfamethoxazole/trimethoprim (STX), and tigecycline (TGC) varied to be sensitive, intermediate, or resistant. The frequency of antimicrobial resistance is shown in Figure 2. Additionally, all isolates had minimum inhibitory concentration MIC > 1024 mg/L for imipenem, while MICs of meropenem ranged from 16 to > 1024 mg/L as shown in Figure 3.

### 2.3. Phenotypic and Genotypic Characterization of Carbapenem-Resistant Isolates

Only 66 isolates (56.41%) were positive for non-specific detection of carbapenemases production by the modified Hodge test (MHT). In contrast, the inhibitor-based tests were more specific by confirmation of carbapenemases production and differentiation between their types. Seventy-nine isolates (67.52%) were positive for MβLs production, and no isolates showed positive results for KPC production or AmpC activity. The remaining isolates (n = 38) did not comply with the criteria of the previously mentioned enzymes but showed resistance to temocillin, giving a presumptive estimation of the presence of the *bla*_OXA-48-like_ genes. The results of both phenotypic tests in comparison with the further molecular screening of carbapenemases genes are shown in Table S2.

The results of molecular screening of the investigated carbapenemases are shown in Table S2. VIM gene was the most prevalent gene amongst the studied genes as it was detected in 99 isolates (84.62%) followed by 88 isolates (75.21%) for NDM gene, 69 isolates (58.97%) for IMP gene, and 34 isolates (29.06%) for OXA-48-like genes. The presence of KPC gene was very limited as it was detected in only 6 isolates (5.13%).

Furthermore, all isolates showed coexistence of at least two carbapenemases under investigation except 4 isolates, namely, 33, 136, 147, and 396, which had VIM gene only; 2 isolates, namely, 16 and 417 had NDM gene only; and 9 isolates, namely, 8, 28,113,127, 151, 346, 356, 378, and 399 had OXA-48-like genes only. On the other hand, only 4 isolates, namely, 137, 142, 410, and 419 were carbapenem-resistant and showed positive MHT but did not show augmentation in the inhibitor-based tests or any positive result via PCR, and this gives an indication that carbapenem resistance in those isolates may be for mechanisms other than carbapenemases production.

The OXA-48-like genes amplicons of the isolates, namely, 151, 303, 311, 383, 399, 405, 409, and 415 were sequenced, and further sequence analysis showed 100% identity for the OXA-48 gene.

### 2.4. Genotyping of Isolates Harboring bla_OXA-48-like_ Genes

**Figure 4:**
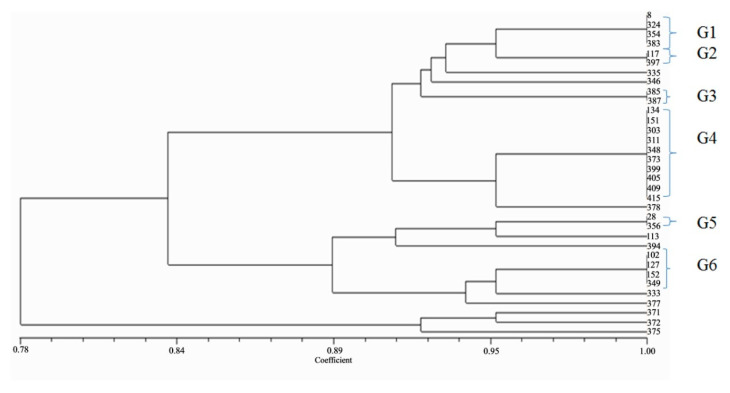
ERIC-PCR dendrogram for 34 isolates harboring *bla*_OXA-48-like_ encoding genes; implemented using NTSYS 2.01 software. The investigated isolates were divided into 16 genotypes. Among them, there were many groups; each represented genetic relatedness. G1(Group 1; isolates: 8, 324, 354, and 383); G2 (Group 2; isolates: 117 and 397); G3 (Group 3; isolates: 385 and 387); G4 (Group 4; isolates: 134, 151, 303, 311, 348, 373, 399, 405, 409, and 415); G5 (Group 5; isolates: 28 and 356), and G6 (Group 6; isolates: 102, 127, 152, and 349). The remaining 10 isolates represent unique patterns (isolates: 113, 333, 335, 346, 371, 372, 375, 377, 378, and 394).

Genotyping of the 34 isolates harboring *bla*_OXA-48-like_ genes was performed by two molecular methods: Enterobacterial Repetitive Intergenic Consensus-Polymerase Chain Reaction (ERIC-PCR) and plasmid profile analysis. Figure 4 shows the ERIC-PCR dendrogram that divided the 34 isolates into 16 genotypes. Among them, there were three major groups: each represented genetic relatedness between its isolates despite their collection from different clinics at different periods. Additionally, 10 isolates showed unique patterns and were not related to each other. On the other hand, the plasmid profile analysis showed variation between isolates. Under experimental conditions, out of 34 isolates, 2 isolates (5.88%) harbored one plasmid, and other isolates harbored many plasmids ranging from 2 to 8 plasmids. The highest number of plasmids was 8 plasmids which were found in one isolate followed by 6 plasmids which were found in another one, representing 2.94% for each. Additionally, there were 11 isolates (32.35%) that had 3 plasmids, followed by 9 isolates (26.47%) that had 5 plasmids, 6 isolates (17.65%) that had 4 plasmids, and 4 isolates (11.76%) that had 2 plasmids. The results showed that the isolates belonging to the same cluster were mostly collected from different sources and at different times.

**Figure 5:**
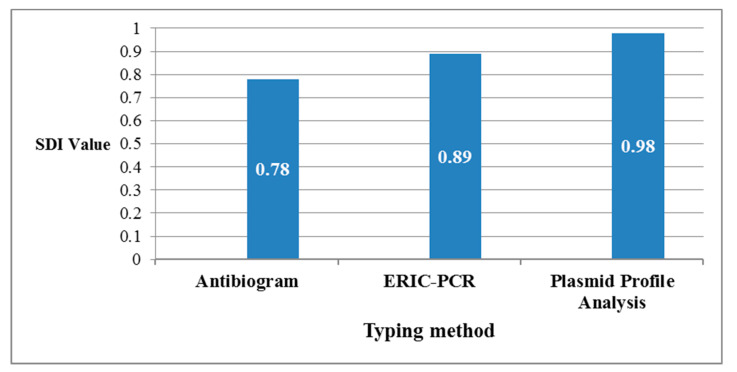
A comparative graph of calculated Simpson’s Diversity Index (SDI) of the applied typing methods to study the relatedness among the 34 *K. pneumoniae* isolates harboring *bla*_OXA-48-like_ genes. SDI values were convergent as they were 0.78, 0.89, and 0.98 for antibiogram, ERIC-PCR, and plasmid profile analysis, respectively.

The Simpson’s Diversity Index was calculated for antibiogram, ERIC-PCR, and plasmid profile analysis giving convergent values of 0.78, 0.89, and 0.98, respectively as shown in Figure 5. The SDI value of the plasmid profile analysis was very close to 1 indicating the maximum diversity that was achieved by this method. The criteria for SDI calculation of the applied typing methods—antibiogram, ERIC-PCR, and plasmid profile analysis—are shown in Tables S3–S5, respectively. A comparative summary of the SDI calculations of the three methods is shown in Table S6.

### 2.5. Transferability of bla_OXA-48_ Gene

The plasmids of five selected isolates, namely, 311, 383, 399, 405, and 409, underwent genetic transfer by chemical transformation. PCR results of transformants showed the absence of *bla*_OXA-48_ gene. Further investigations were performed for two selected transformants, namely, 383 and 409, by an antimicrobial susceptibility test and PCR. The results showed reduced susceptibility to different β-lactams including carbapenems, sulfamethoxazole/trimethoprim, and tigecycline in comparison with *Escherichia coli* DH5α as shown in Table 1. Although the isolate 383 had *bla*_NDM_, *bla*_VIM_, and *bla*_IMP_ encoding genes and the isolate 409 had *bla*_NDM_ and *bla*_VIM_ encoding genes, only *bla*_VIM_ encoding gene was detected by colony PCR for both isolates transformants. This explains the successful transfer of such gene.

### 2.6. Assessment of Biofilm Formation and Detection of Adhesion Encoding Genes in Carbapenem-Resistant K. pneumoniae Isolates

The quantitative biofilm formation assay resulted in OD_600_ values ranging from 0.2 to 1.1. All isolates were classified as biofilm formers but with different capacities. The majority of the isolates were weak biofilm formers and were represented by 82 isolates (70.09%), followed by 34 isolates as moderate formers (29.06%), and only one isolate was classified as a strong biofilm former (0.85%). Furthermore, the PCR screening of the isolates showed the prevalence of the two adhesion encoding genes in most isolates. Out of 117 isolates, *fimH-1* gene was found in 108 isolates (92.31%) and *mrkD* gene was found in 115 isolates (98.29%). Additionally, 106 isolates (90.60%) represented the coexistence of both genes as shown in Table S7.

### 2.7. The Correlation between Non-Susceptibility Pattern and Biofilm Formation Category of Carbapenem-Resistant K. pneumoniae Isolates

As shown in Table 2, there is no significant correlation between non-susceptibility patterns and biofilm formation categories among carbapenem-resistant isolates. All *p*-values were > 0.05, and this was considered statistically insignificant.

## 3. Discussion

The dissemination of carbapenemases encoding genes in Egypt, especially in healthcare settings, is of great concern in several mapping studies [20,21,22]. The prevalence of such genes in Egypt could be attributed to considerable reasons that are well-identified globally. For example, hospital environments are considered as a pool for the dissemination of such genes through the horizontal gene transfer that enables their spread via mobile genetic elements such as plasmids [23,24]. Additionally, plasmids may carry more than one resistance gene, and this leads to resistance to diverse antimicrobial classes and as a result limited therapeutic options [25].

Although the transferability of the OXA-48 gene of our selected isolates failed and this was compliant with Skalova et al. trials [26], the reduced transformants susceptibility to most antimicrobials confirms the genetic transfer risk. Additionally, over-the-counter prescription and the misuse of antimicrobials participate in the exacerbation of the antimicrobial resistance problem, especially in Egypt [27,28].

In parallel, the investigated isolates in our study showed increased resistance to various antimicrobial agents and also high MIC values for imipenem and meropenem. This may be markedly attributed to various resistance mechanisms that led to resistance to most antimicrobial classes. Additionally, only 15 isolates (12.82%) of the total investigated isolates were of sole carbapenemase, indicating remarkable coexistence. This finding is most consistent with studies from Egyptian hospitals and unlike the study by Argente et al. in which *bla*_OXA-48_ gene was the only detected carbapenemase among the tested isolates [29]. This reflects the diverse regional distribution of carbapenemases.

In the current study, the detection and typing of carbapenemases depended on the merge between the phenotypic and molecular methods, and this is in consistency with the study by Karampatakis et al. [30]. The application of the molecular methods in the detection of resistance genes and further typing of isolates is considered as a gold standard tool in the field of epidemiology, and this appears on two occasions in this study; Firstly, the disagreement between phenotypic and genotypic methods for the detection of *bla*_KPC_ encoding gene which was detected in only 6 isolates by PCR. In this case, the genotypic methods act as an alarming tool for the silent dissemination of such gene which is absent phenotypically but likely to be successfully expressed under certain conditions [31]. Secondly, there is no phenotypic test for the direct detection of *bla*_OXA-48-like_ genes. Resistance to temocillin is considered as a stepwise prediction of them, since other resistance mechanisms may confer temocillin resistance [32].

In our work, we applied ERIC-PCR and plasmid profile analysis, besides the previously determined antibiogram for the characterization of isolates harboring *bla*_OXA-48-like_ genes. The discriminatory power of the applied typing methods (antibiogram, ERIC-PCR, and plasmid profile analysis) was approximated according to the SDI calculations, while the highest diversity was represented by plasmid profile analysis (SDI = 0.98). The three typing methods are different in their concepts, and this leads to variation in the clonality of the isolates among the three methods.

The interplay between the antimicrobial resistance and biofilm formation was studied from different sides. On the one hand, resistance genes encoded on some plasmids regulate the expression of fimbrial genes, which are among the essentials of biofilm formation. On the other hand, the plasmid horizontal gene transfer is enhanced in biofilms more than in planktonic cells [33]. In our work, all investigated isolates were carbapenem-resistant and showed biofilm formation ability which was various and independent on the resistance profile of those isolates. It is worth mentioning that despite the absence of such correlation, having 100% of isolates as biofilm formers with the high resistance pattern is considered a troublesome feature of the studied isolates.

Our results were in concordance with the study by El Fertas-Aissani et al. on the prevalence of *fimH-1* and *mrkD* adhesion encoding genes, giving an indication of their conservation in *K. pneumoniae* pathogen [34]. Furthermore, the prevalence of those two adhesion genes and the weak biofilm patterns among the isolates may suggest that the strong biofilm formation may need factors other than fimbrial adhesion genes.

Three isolates in our study, namely, 349, 354, and 375, represented unique findings, as they were resistant to all applied antimicrobials except tigecycline. Additionally, they showed positive results for the MHT, all carbapenemases (*bla*_KPC_, *bla*_NDM_, *bla*_VIM_, *bla*_IMP_, and *bla*_OXA-48-like_) encoding genes, and for *fimH-1* and *mrkD* encoding genes. Although our applied typing methods are relatively simple and of low cost, the results of the three isolates encourage the adoption of other typing methods such as whole-genome sequencing (WGS) [35]. Whole-genome sequencing provides massive data for the determination of the sequence type, plasmid replicon, antimicrobial resistance determinants, and virulence genes. Additionally, *K. pneumoniae* was of special concern for the newly emerged bioinformatics interfaces such as Kaptive Web [36] and KlebNet [37]. Additionally, we are moving globally toward the extended drug resistance with some reported cases of alarmingly pan-drug-resistant isolates [38], research is directed toward finding alternatives for combating antibiotic resistance. The alternative strategies may include antibiotic combinations [39], phage therapy [40], vaccine development [41], natural products [42], revival of classical antibiotics [43], or repurposing non-antimicrobials for targeting pathogens [44].

Finally, the applied infection control measures and awareness campaigns may not be enough to combat the problem of antimicrobial resistance, especially in a developing country such as Egypt. Among the important strategies that are worth emphasizing is launching collaborative platforms between Egyptian healthcare institutes and other international ones for networking and gaining accurate knowledge about the possible patterns of antimicrobial resistance that could be easily disseminated globally [45,46].

## 4. Materials and Methods

### 4.1. Isolates Collection and Confirmation

A total of 117 archival non-duplicate and non-consecutive isolates of carbapenem-non-susceptible (resistant or intermediate) *K. pneumoniae* were recovered from clinical specimens. The specimens were collected and processed by dedicated healthcare workers. Furthermore, the specimens were collected from patients admitted to different departments of Kasr Al-Ainy Hospital in Cairo, Egypt, from September 2014 to December 2016. The study was approved by the Ethics Committee of the Faculty of Pharmacy, Al-Azhar University (Girls’ branch). The ethical approval code is SMR/2015.

The collected isolates were obtained from the diagnostic routine work of the Clinical Pathology Department of Kasr Al-Ainy Hospital without direct contact with the patient or their related personal data. All laboratory techniques and procedures of isolation, identification, and storage of the included isolates in the current study were performed according to the standard microbiological techniques.

The isolates were previously identified using conventional microbiological methods which included Gram staining, cultural characteristics on MacCkonky’s agar, and biochemical testing such as growth on Triple Sugar Iron agar (TSI) and Indole, Methyl red, Voges-Proskauer, and Citrate utilization (IMViC) test. The identification of *K. pneumoniae* isolates was confirmed using Matrix-Assisted Laser Desorption/Ionization Time-Of-Flight Mass Spectrometry (MALDI-TOF/MS) (Vitek MS; BioMérieux, Inc., Marcy-l'Etoile, France).

### 4.2. Antimicrobial Susceptibility Testing

Antimicrobial susceptibility testing was carried out by disc diffusion according to the Kirby-Bauer method. The isolates were tested against the following antimicrobial agents: amoxicillin/clavulanic acid (20/10 µg), piperacillin/tazobactam (100/10 µg), cefoxitin (30 µg), cefotaxime (30 µg), cefotaxime/clavulanic acid (30/10 µg), ceftazidime (30 µg), ceftazidime/clavulanic acid (30/10 µg), cefepime (30 µg), aztreonam (30 µg), imipenem (10 µg), meropenem (10 µg), ertapenem (10 µg), gentamicin (10 µg), amikacin (30 µg), ciprofloxacin (5 µg), sulfamethoxazole/trimethoprim (1.25/23.75 μg),and tigecycline (15 µg), (Oxoid, Basingstoke, UK). *E. coli* ATCC^®^ 25922, *E. coli* ATCC^®^ 35218, and *P.aeruginosa* ATCC^®^ 27853 were used as quality control strains. The minimum inhibitory concentrations (MIC) of meropenem (commercially available as Meronem^TM^ (500 mg) IV, AstraZeneca, Macclesfield, UK, Ltd.) and imipenem (commercially available as Tienam ^TM^ (500 mg) Injection, Merck Sharp & Dohme BV, Haarlem, The Netherlands) were determined by a microdilution method. The antimicrobial-free media was used as a positive control and the un-inoculated media was used as a negative control. The results were interpreted according to the Clinical and Laboratory Standards Institute guidelines, CLSI (M100-S26, 2016) [47]. Tigecycline interpretive criteria were according to the FDA recommendations. http://www.accessdata.fda.gov/drugsatfda_docs/label/2009/021821s016lbl.pdf. The interpretation criteria of the different antimicrobial agents are mentioned in Table S8.

### 4.3. Phenotypic Confirmation of Carbapenemases Production

Non-specific screening of carbapenemases production was performed by the modified Hodge test (MHT) according to guidelines of CLSI [47]. The formation of cloverleaf-like indentation of grown *E. coli* ATCC^®^ 25922 along the tested isolate is an indication of the positive result. *K. pneumoniae* ATCC^®^ BAA-1705 was used as a positive control, and *K. pneumoniae* ATCC^®^ BAA-1706 was used as a negative control. Moreover, the inhibitor-based tests were performed as another phenotypic approach for further differentiation between carbapenemases producing isolates. The inhibitor-based tests were based on the inactivation of carbapenemases by combining some substrates independently with meropenem disc. For example, EDTA as metallo-β-lactamases (MβLs) inhibitor, boronic acid as KPC inhibitor, and cloxacillin as AmpC inhibitor [48]. The inhibitor-based tests were performed using MASTDISCS^TM^ ID Carbapenemase Detection Disc Set D70C (MAST group, Merseyside, UK). The set consists of four different discs as shown in Table 3. The interpretation of the tests depends on the comparison of the inhibition zone of the meropenem disc with the inhibition zone of the disc containing meropenem combined with the carbapenemases inhibitor. An additional Temocillin disc (30 µg) was applied for obtaining a preliminary indication of the *bla*_OXA-48-like_ production until performing further genotypic confirmatory tests.

### 4.4. Molecular Detection of Carbapenemases Encoding Genes

Total DNA of the isolates was extracted by the boiling method according to Vaneechoutte et al. [49]. Uniplex PCR reactions were performed for detection of *bla*_KPC_, *bla*_NDM_, *bla*_VIM_, *bla*_IMP_, and *bla*_oxa-48-like_ encoding genes with primers listed in Table S9 [50,51,52]. The primer specificity was checked against sequences retrieved from GenBank sequence databases using the National Center for Biotechnology Information/Basic Local Alignment Search Tool (NCBI/BLAST), allowing the detection of several variants of each carbapenemase. The primers were synthesized by Eurofins MWG Operon (Ebersberg, Germany). The applied profile was according to the criteria that were mentioned in primer references except for adjustment of annealing temperature to be 56 °C. The positive controls were obtained from the Clinical and Chemical Pathology Department laboratory collection (Kasr Al-Ainy hospital, Cairo, Egypt) and represented by positive strains that harbored investigated carbapenemases encoding genes and had been previously sequenced. The negative control was a template-free master mix.

The resulted PCR amplicons were electrophorized on 0.8% gel alongside a 1-kb ladder. Selected *bla*_OXA-48-like_ gene amplicons, namely, 151, 303, 311, 383, 399, 405, 409, and 415, were sequenced for further investigation using pre-OXA-48 primers targeting the gene and its flanking regions [53].

PCR products were submitted for purification and sequencing in both directions via (Solgent Co. Ltd., Daejeon, Korea). The resulted DNA sequences were trimmed using Geneious Software v.8.1.6 (www.geneious.com) and compared to the Genbank sequence database using the BlASTn tool at https://blast.ncbi.nlm.nih.gov/Blast.cgi.

### 4.5. Genotyping of Isolates Harboring bla_OXA-48-like_ Genes

Thirty-four isolates were confirmed either by inhibitor-based tests and/or PCR as harboring *bla*_OXA-48-like_ genes. These isolates were genotyped by two methods: the first one is the Enterobacterial Repetitive Intergenic Consensus-Polymerase Chain Reaction (ERIC-PCR) which aimed to determine the clonal relatedness between the investigated isolates by detection of specific conserved sequences among them [54]. The second typing method is the plasmid profile analysis which depends on the investigation of the isolate plasmids and characterization of their profile [55].

#### 4.5.1. Enterobacterial Repetitive Intergenic Consensus (ERIC-PCR)

The genomic DNA was extracted using a GeneJet Genomic DNA Purification Kit (K0721, Thermo Scientific, Rockford, IL, USA) according to the manufacturer’s protocol. The investigated isolates were genotyped by ERIC-PCR using ERIC-2 primer (5’ AAGTAAGTGACTGGGGTGAGCG 3’) [56]. The PCR profile was according to Abdulall et al. [57] with slight modifications as follows: initial denaturation at 95 °C for 5 min, followed by 35 cycles of denaturation at 95 °C for 1 min, annealing at 45 °C for 1 min, and extension at 72 °C for 8 min, with a final extension at 72 °C for 10 min. Aliquots of PCR amplicons were electrophorized alongside a 10-kb Ladder. The interpretation of the results depended on binary data by which the positive and negative amplifications were assigned as 1/0, respectively. Cluster analysis was performed using NTSYS 2.01 software and the schematic dendrogram was built using the Unweighted Pair-Group Method using Arithmetic Mean (UPGMA).

#### 4.5.2. Plasmid Profile Analysis

The isolate plasmids were extracted by a QIAprep^®^ Spin Miniprep Kit according to the manufacturer’s protocol. Extracted plasmids were electrophorized, and the generated patterns were judged visually and considered of distinct type if they showed a single band of different size, according to the ESGEM recommendations [15].

### 4.6. Calculation of the Discriminatory Power of Different Applied Typing Methods

The discriminatory power of the applied typing methods (antibiogram, ERIC-PCR, and plasmid profile analysis) among the 34 isolates was assessed using Simpson’s Diversity Index (SDI). The concept of discrimination based on if there are two unrelated isolates, which were sampled from a test population, they will be probably found in different typing groups [58]. The SDI calculation depended on the following equation $D=1-\frac{\sum \;n\left(n-1\right)}{\;N\left(N-1\right)}$, where D is the diversity index, n is the number of individuals of each species, and N is the total number of individuals of all species. The SDI ranges from 0, which indicates the identical types of the tested isolates, to 1, which indicates the maximum diversity among them.

### 4.7. Genetic Transfer of bla_OXA-48_ Gene by Transformation

The acquisition of resistance determinants harbored by the isolate plasmids was assessed by transformation. The preparation and transformation of competent *E. coli* DH5α cells were performed using the CaCl_2_ method according to Cohen et al. [59]. The genetic transfer of *bla*_OXA-48_ gene was assessed for the plasmids of five selected isolates, namely, 311, 383, 399, 405, and 409. The transformants were selected on LB agar containing ampicillin (50 mg/L) and also on LB agar containing meropenem (0.5 mg/L). The plasmids of transformants were extracted and underwent PCR for the detection of the *bla*_OXA-48_ gene.

### 4.8. Quantitative Biofilm Formation Assay for the Carbapenem-Resistant Isolates by Microtitre Plate Method

The quantitative assessment of biofilm formation for the 117 carbapenem-resistant isolates was performed by the crystal violet staining assay according to O’Toole protocol with slight modifications [60]. Briefly, overnight culture of each isolate was grown in Luria-Bertani broth, and then was diluted 1:100 into fresh LB medium. One hundred microliters of the isolate dilution was added per well in a flat-bottomed 96 well microtitre plate. After overnight incubation at 37 °C, the medium was discarded, and the wells were washed twice by adding 100 µL of distilled water in each well for further removal of unattached cells and media. The plate was allowed to dry, stained by adding 125 µL of 0.1% crystal violet to each well, and incubated at room temperature for 15 min. The crystal violet was discarded and the plate was washed four times for removing excess stain and allowed to dry. The stained biofilms were solubilized by adding 125 µL of 30% acetic acid to each well and incubated for 15 min at room temperature. The solubilized stain of each well was transferred to a new microtiter plate. The optical density of each well was measured at 600 nm (OD_600_) using a microplate reader (Stat Fax^®^ 2100, Awareness Technology Inc., Palm City, FL, USA). The experiments were carried out three times in triplicates; the readings of each isolate in each plate were averaged and compared for further interpretation. The positive control was *K. pneumoniae* ATCC^®^ 700603 and the negative control was un-inoculated LB media. The interpretation of the results was according to the recommendations of Stepanović et al. recommendations [61].

### 4.9. Molecular Detection of Type 1 (fimH-1) and Type 3 (mrkD) Adhesion Encoding Genes

The carbapenem-resistant isolates were screened for the presence of *fimH-1* and *mrkD* encoding genes by uniplex PCR using primers listed in Table S9 [34,62]. The applied profile was as follows: initial denaturation at 94 °C for 4 min followed by 30 cycles of denaturation at 94 °C for 30 s, annealing at 55 °C for 40 s, extension at 72 °C for 1 min, and a final extension at 72 °C for 10 min. The positive controls were obtained from the MTI University Microbiology Laboratory archive, Egypt, and represented by positive strains that harbored investigated adhesion genes and had been previously sequenced. The negative control was a template-free master mix.

### 4.10. Statistical Analysis

Statistical analysis was performed using GraphPad Prism software (San Diego, CA, USA). The description of qualitative data was as a frequency percentage. The chi-square or Fisher’s exact test was used for comparing categorical variables. The results were considered statistically significant when *p*-value < 0.05. Spearman’s test was applied for the determination of the correlation between the biofilm formation category and antimicrobial resistance profile of all isolates.

## 5. Conclusions 

In our work, we studied the prevalence of the ”big five” carbapenemases encoding genes, (*bla*_KPC_, *bla*_NDM_, *bla*_VIM_, *bla*_IMP_, and *bla*_OXA-48-like_) among carbapenem-resistant *K. pneumoniae* isolated from a tertiary hospital in Egypt. The tested carbapenem-resistant *K. pneumoniae* isolates showed a remarkable coexistence of different investigated carbapenemases. Additionally, we shed light on *bla*_OXA-48-like_ genes which represented about one-third of our isolates. The assessment of the simple epidemiological typing methods—antibiogram, ERIC-PCR, and plasmid profile analysis—showed their convergent discriminatory power. Further investigations showed the biofilm formation ability of all isolates with the prevalence of adhesion genes (*fim-H* and *mrkD*). The presence of a high resistance pattern with biofilm formation ability represents a worrisome feature of a superbug such as *K. pneumoniae* in the Egyptian healthcare setting. Thus, there is an urgent need for further strategies for combating resistance, in-depth studies for the resistance genes and their transfer, and also other characteristics such as biofilm formation which all strongly improve the ability of any pathogen to cause high rates of morbidity and mortality. 

## Figures and Tables

**Table 1:** Comparison of antimicrobial susceptibilities between *E. coli* DH5α and two transformants.

Isolate	AUG	TZP	FOX	CTX	CTC	CAZ	CZC	CPM	ATM	MEM	ETR	GN	AK	CIP	STX	TGC
DH5α	R	S	S	S	S	S	S	S	S	S	S	S	S	S	S	S
transformant 383	R	R	R	R	R	R	R	R	R	I	R	S	S	S	R	R
transformant 409	R	R	R	R	R	R	R	R	R	R	R	S	S	S	R	R

**Table 2:** Correlation between non-susceptibility pattern and biofilm formation.

Non-Susceptibility Pattern		Biofilm Pattern			*p*-Value (>0.05)
		Strong	Moderate	Weak	
**A**	**Non-susceptible to all antimicrobials.**	1	21	52	0.3333
B	Non-susceptible to all Except AK.	0	0	2	0.6667
C	Non-susceptible to all Except GN	0	0	4	0.6667
D	Non-susceptible to all Except CIP	0	0	1	0.6667
E	Non-susceptible to all Except STX	0	3	2	>0.9999
F	Non-susceptible to all Except TGC	0	7	13	0.3333
G	Non-susceptible to all Except ATM & AK	0	1	0	>0.9999
H	Non-susceptible to all Except GN & AK	0	0	2	0.6667
I	Non-susceptible to all Except TGC & AK	0	1	0	>0.9999
J	Non-susceptible to all Except STX & TGC	0	1	0	>0.9999
K	Non-susceptible to all Except GN & TGC	0	0	3	0.6667
L	Non-susceptible to all Except GN, CIP & STX	0	0	1	0.6667
M	Non-susceptible to all Except GN, STX & TGC	0	0	1	0.6667
N	Non-susceptible to all Except GN, CIP, STX & TGC	0	0	1	0.6667

**Table 3:** The interpretative criteria of applied inhibitor-based tests.

Disc	Content	Interpretative Criteria
	Meropenem (10 µg).	Confirmation of Carbapenem-non-susceptibility.
B	Meropenem + MβL inhibitor.	Confirmation of MβL production (if only B-A ≥ 5 mm).
C	Meropenem + KPC inhibitor.	Confirmation of KPC production (if only C-A ≥ 4 mm).
D	Meropenem + AmpC inhibitor.	Confirmation of AmpC+ porin loss (if both C-A ≥ 4 mm and D-A ≥ 5 mm)
TEM	Temocillin (30 µg).	Indicative for OXA-48 (if there was no synergy detected and the inhibition zone of Temocillin was <11 mm)

## Supplementary Materials

The following are available online https://www.mdpi.com/2079-6382/9/12/852/s1. Table S1: Antimicrobial susceptibility patterns of *Klebsiella pneumoniae* isolates by disc diffusion method; Table S2: Results of modified Hodge test, inhibitor-based tests, and genotypic detection of investigated carbapenemases; Table S3: Simpson’s Diversity Index calculations for antibiogram; Table S4: Simpson’s Diversity Index calculations for ERIC-PCR; Table S5: Simpson’s Diversity Index calculations for plasmid profile analysis; Table S6: A comparative summary of SDI calculations of the three typing methods; Table S7: Distribution of *fimH-1* and *mrkD* adhesion genes among the carbapenem-resistant isolates; Table S8: The applied antimicrobial agents, their classes, and their interpretive categories; Table S9: Primer sequences and the expected amplicon sizes of investigated genes.

## References

[1] O’Neill J.I.M. (2014). Antimicrobial resistance: Tackling a crisis for the health and wealth of nations. Rev. Antimicrob. Resist..

[2] World Health Organization (2014). Antimicrobial Resistance: Global Report on Surveillance.

[3] Centers for Disease Control and Prevention (2018). About Antimicrobial Resistance. https://www.cdc.gov/drugresistance/about.html.

[4] World Bank (2017). Drug-Resistant Infections: A Threat to Our Economic Future.

[5] Rice L.B. (2010). Progress and Challenges in Implementing the Research on ESKAPE Pathogens. Infect. Control. Hosp. Epidemiol..

[6] World Health Organization (2017). Global Priority List of Antibiotic-Resistant Bacteria to Guide Research, Discovery, and Development of New Antibiotics. http://www.who.int/medicines/publications/global-priority-list-antibiotic-resistant-bacteria/en/.

[7] Codjoe F.S., Donkor E.S. (2017). Carbapenem Resistance: A Review. Med. Sci..

[8] Halat D.H., Moubareck C.A. (2020). The Current Burden of Carbapenemases: Review of Significant Properties and Dissemination among Gram-Negative Bacteria. Antibiotics.

[9] Bratu S., Landman D., Haag R., Recco R., Eramo A., Alam M., Quale J. (2005). Rapid Spread of Carbapenem-Resistant Klebsiella pneumoniae in New York City. Arch. Intern. Med..

[10] Zhao W.H., Hu Z.Q. (2015). Acquired metallo-β-lactamases and their genetic association with class 1 integrons and ISCRelements in Gram-negative bacteria. Futur. Microbiol..

[11] Poirel L., Potron A., Nordmann P. (2012). OXA-48-like carbapenemases: The phantom menace. J. Antimicrob. Chemother..

[12] Wyres K.L., Lam M.M.C., Holt K.E. (2020). Population genomics of Klebsiella pneumoniae. Nat. Rev. Genet..

[13] Pitout J., Peirano G., Kock M.M., Strydom K.A., Matsumura Y. (2019). The Global Ascendency of OXA-48-Type Carbapenemases. Clin. Microbiol. Rev..

[14] Potron A., Poirel L., Nordmann P. (2013). Derepressed Transfer Properties Leading to the Efficient Spread of the Plasmid Encoding Carbapenemase OXA-48. Antimicrob. Agents Chemother..

[15] Van Belkum A., Tassios P., Dijkshoorn L., Haeggman S., Cookson B., Fry N., Fussing V., Green J., Feil E., Gerner-Smidt P. (2007). Guidelines for the validation and application of typing methods for use in bacterial epidemiology. Clin. Microbiol. Infect..

[16] Vert M., Doi Y., Hellwich K.H., Hess M., Hodge P.E., Kubisa P., Rinaudo M., Schué F. (2012). Terminology for biorelated polymers and applications (IUPAC Recommendations 2012). Pure Appl. Chem..

[17] Vuotto C., Longo F., Balice M.P., Donelli G., Varaldo P.E. (2014). Antibiotic Resistance Related to Biofilm Formation in *Klebsiella pneumoniae*. Pathogens.

[18] Alcántar-Curiel M.D., Blackburn D., Saldaña Z., Gayosso-Vázquez C., Iovine N.M., De La Cruz M.A., Girón J.A. (2013). Multi-functional analysis ofKlebsiella pneumoniaefimbrial types in adherence and biofilm formation. Virulence.

[19] Magiorakos A.P., Srinivasan A., Carey R., Carmeli Y., Falagas M., Giske C., Harbarth S.J., Hindler J., Kahlmeter G., Olsson-Liljequist B. (2012). Multidrug-resistant, extensively drug-resistant and pandrug-resistant bacteria: An international expert proposal for interim standard definitions for acquired resistance. Clin. Microbiol. Infect..

[20] Manenzhe R.I., Zar H.J., Nicol M.P., Kaba M. (2015). The spread of carbapenemase-producing bacteria in Africa: A systematic review. J. Antimicrob. Chemother..

[21] Sekyere J.O., Govinden U., Essack S. (2016). The Molecular Epidemiology and Genetic Environment of Carbapenemases Detected in Africa. Microb. Drug Resist..

[22] Touati A., Mairi A. (2020). Epidemiology of Carbapenemase-producing Enterobacterales in the Middle East: A systematic review. Expert Rev. Anti-Infect. Ther..

[23] Yan Z., Zhou Y., Du M., Bai Y., Liu B., Gong M., Song H., Tong Y., Liu Y. (2019). Prospective investigation of carbapenem-resistant *Klebsiella pneumonia* transmission among the staff, environment and patients in five major intensive care units, Beijing. J. Hosp. Infect..

[24] Lerminiaux N.A., Cameron A.D. (2019). Horizontal transfer of antibiotic resistance genes in clinical environments. Can. J. Microbiol..

[25] Carattoli A. (2013). Plasmids and the spread of resistance. Int. J. Med. Microbiol..

[26] Skalova A., Chudejova K., Rotova V., Medvecky M., Studentova V., Chudackova E., Lavicka P., Bergerova T., Jakubu V., Zemlickova H. (2016). Molecular Characterization of OXA-48-Like-Producing Enterobacteriaceae in the Czech Republic and Evidence for Horizontal Transfer of pOXA-48-Like Plasmids. Antimicrob. Agents Chemother..

[27] Laxminarayan R., Duse A., Wattal C., Zaidi A.K.M., Wertheim H., Sumpradit N., Vlieghe E., Hara G.L., Gould I.M., Goossens H. (2013). Antibiotic Resistance—The Need for Global Solutions. Lancet Infect. Dis..

[28] Zakaa El-din M., Samy F., Mohamed A., Hamdy F., Yasser S., Ehab M. (2018). Egyptian community pharmacists’ attitudes and practices towards antibiotic dispensing and antibiotic resistance; a cross-sectional survey in Greater Cairo. Curr. Med. Res. Opin..

[29] Argente M., Miró E., Martí C., Vilamala A., Alonso-Tarrés C., Ballester F., Calderon A., Galles C., Gasós A., Mirelis B. (2019). Molecular characterization of OXA-48 carbapenemase-producing *Klebsiella pneumoniae* strains after a carbapenem resistance increase in Catalonia. Enferm. Infecc. Microbiol. Clínica.

[30] Karampatakis T., Geladari A., Politi L., Antachopoulos C., Iosifidis E., Tsiatsiou O., Karyoti A., Papanikolaou V., Tsakris A., Roilides E. (2017). Cluster-distinguishing genotypic and phenotypic diversity of carbapenem-resistant Gram-negative bacteria in solid-organ transplantation patients: A comparative study. J. Med. Microbiol..

[31] Azimi L., Talebi M., Owlia P., Pourshafie M.-R., Najafi M., Lari E.R., Lari A.R., Najafi M. (2016). Tracing of false negative results in phenotypic methods for identification of carbapenemase by Real-time PCR. Gene.

[32] (2017). EUCAST Subcommittee for Detection of Resistance Mechanisms and Specific Resistances of Clinical and/or Epidemiological Importance. http://www.eucast.org/fileadmin/src/media/PDFs/EUCAST_files/Resistance_mechanisms/EUCAST_detection_of_resistance_mechanisms_170711.pdf.

[33] Madsen J.S., Burmølle M., Hansen L.H., Sørensen S.J. (2012). The interconnection between biofilm formation and horizontal gene transfer. FEMS Immunol. Med. Microbiol..

[34] El Fertas-Aissani R., Messai Y., Alouache S., Bakour R. (2013). Virulence profiles and antibiotic susceptibility patterns of Klebsiella pneumoniae strains isolated from different clinical specimens. Pathol. Biol..

[35] Köser C.U., Ellington M.J., Cartwright E.J.P., Gillespie S.H., Brown N.M., Farrington M., Holden M.T.G., Dougan G., Bentley S.D., Parkhill J. (2012). Routine Use of Microbial Whole Genome Sequencing in Diagnostic and Public Health Microbiology. PLoS Pathog..

[36] Wick R.R., Heinz E., Holt K.E., Wyres K.L. (2018). Kaptive Web: User-Friendly Capsule and Lipopolysaccharide Serotype Prediction for Klebsiella Genomes. J. Clin. Microbiol..

[37] Lee M., Pinto N.A., Kim C.Y., Yang S., D’Souza R., Yong D., Lee I. (2019). Network Integrative Genomic and Transcriptomic Analysis of Carbapenem-Resistant Klebsiella pneumoniae Strains Identifies Genes for Antibiotic Resistance and Virulence. mSystems.

[38] Zowawi H.M., Forde B.M., Alfaresi M., Alzarouni A., Farahat Y., Chong T.-M., Yin W.F., Chan K.-G., Li J., Schembri M.A. (2015). Stepwise evolution of pandrug-resistance in *Klebsiella pneumoniae*. Sci. Rep..

[39] Daikos G.L., Tsaousi S., Tzouvelekis L.S., Anyfantis I., Psichogiou M., Argyropoulou A., Stefanou I., Sypsa V., Miriagou V., Nepka M. (2014). Carbapenemase-Producing *Klebsiella pneumoniae* Bloodstream Infections: Lowering Mortality by Antibiotic Combination Schemes and the Role of Carbapenems. Antimicrob. Agents Chemother..

[40] Kęsik-Szeloch A., Drulis-Kawa Z., Weber-Dąbrowska B., Kassner J., Majkowska-Skrobek G., Augustyniak D., Łusiak-Szelachowska M., Żaczek M., Górski A., Kropinski A.M. (2013). Characterising the biology of novel lytic bacteriophages infecting multidrug resistant *Klebsiella pneumoniae*. Virol. J..

[41] Ahmad T.A., El-Sayed L.H., Haroun M., Hussein A.A., El Ashry E.S.H. (2012). Development of immunization trials against *Klebsiella pneumoniae*. Vaccine.

[42] Somboro A.M., Sekyere J.O., Amoako D.G., Kumalo H.M., Khan R., Bester L.A., Essack S. (2018). In vitro potentiation of carbapenems with tannic acid against carbapenemase-producing enterobacteriaceae: Exploring natural products as potential carbapenemase inhibitors. J. Appl. Microbiol..

[43] Cassir N., Rolain J.-M., Brouqui P. (2014). A new strategy to fight antimicrobial resistance: The revival of old antibiotics. Front. Microbiol..

[44] Younis W., Abdelkhalek A., Mayhoub A.S., Seleem M.N. (2017). In Vitro Screening of an FDA-Approved Library Against ESKAPE Pathogens. Curr. Pharm. Des..

[45] Rosenthal V.D., Bat-Erdene I., Gupta D., Belkebir S., Rajhans P., Zand F., Myatra S.N., Afeef M., Tanzi V.L., Muralidharan S. (2020). Six-year multicenter study on short-term peripheral venous catheters-related bloodstream infection rates in 727 intensive care units of 268 hospitals in 141 cities of 42 countries of Africa, the Americas, Eastern Mediterranean, Europe, South East Asia, and Western Pacific Regions: International Nosocomial Infection Control Consortium (INICC) findings. Infect. Control Hosp. Epidemiol..

[46] Al-Hassan L., Roemer-Mahler A., Price J., Islam J., El-Mahallawy H., Higgins P.G., Hussein A.F.A., Roca I., Newport M. (2020). The TACTIC experience: Establishing an international, interdisciplinary network to tackle antimicrobial resistance. J. Med. Microbiol..

[47] Clinical and Laboratory Standards Institute (2016). Performance Standards for Antimicrobial Susceptibility Testing. CLSI Document M100-S26.

[48] Lutgring J.D., Limbago B. (2016). The Problem of Carbapenemase-Producing-Carbapenem-Resistant-Enterobacteriaceae Detection. J. Clin. Microbiol..

[49] Vaneechoutte M., Dijkshoorn L., Tjernberg I., Elaichouni A., De Vos P., Claeys G., Verschraegen G. (1995). Identification of Acinetobacter genomic species by amplified ribosomal DNA restriction analysis. J. Clin. Microbiol..

[50] Akpaka P.E., Swanston W.H., Ihemere H.N., Correa A., Torres J.A., Tafur J.D., Montealegre M.C., Quinn J.P., Villegas M.V. (2009). Emergence of KPC-Producing Pseudomonas aeruginosa in Trinidad and Tobago. J. Clin. Microbiol..

[51] Poirel L., Walsh T.R., Cuvillier V., Nordmann P. (2011). Multiplex PCR for detection of acquired carbapenemase genes. Diagn. Microbiol. Infect. Dis..

[52] Aktaş Z., Kayacan C.B., Schneider I., Can B., Midilli K., Bauernfeind A. (2008). Carbapenem-Hydrolyzing Oxacillinase, OXA-48, Persists in *Klebsiella pneumoniae* in Istanbul, Turkey. Chemotherapy.

[53] Potron A., Nordmann P., Lafeuille E., Al Maskari Z., Al Rashdi F., Poirel L. (2011). Characterization of OXA-181, a Carbapenem-Hydrolyzing Class D β-Lactamase from *Klebsiella pneumoniae*. Antimicrob. Agents Chemother..

[54] Wilson L.A. (2006). Sharp, P.M. Enterobacterial Repetitive Intergenic Consensus (ERIC) Sequences in Escherichia coli: Evolution and Implications for ERIC-PCR. Mol. Biol. Evol..

[55] Mayer L.W. (1988). Use of plasmid profiles in epidemiologic surveillance of disease outbreaks and in tracing the transmission of antibiotic resistance. Clin. Microbiol. Rev..

[56] Versalovic J., Koeuth T., Lupski J.R. (1991). Distribution of repetitive DNA sequences in eubacteria and application to finerpriting of bacterial enomes. Nucleic Acids Res..

[57] Abdulall A.K., Tawfick M.M., El-Manakhly A.R., El-Kholy A. (2018). Carbapenem-resistant Gram-negative bacteria associated with catheter-related bloodstream infections in three intensive care units in Egypt. Eur. J. Clin. Microbiol. Infect. Dis..

[58] Hunter P.R., Gaston M.A. (1988). Numerical index of the discriminatory ability of typing systems: An application of Simpson’s index of diversity. J. Clin. Microbiol..

[59] Cohen S.N., Chang A.C.Y., Hsu L. (1972). Nonchromosomal Antibiotic Resistance in Bacteria: Genetic Transformation of *Escherichia coli* by R-Factor DNA. Proc. Natl. Acad. Sci. USA.

[60] O’Toole G.A. (2011). Microtiter Dish Biofilm Formation Assay. J. Vis. Exp..

[61] Stepanović S., Vuković D., Hola V., Bonaventura G.D., Djukić S., Ćirković I., Ruzicka F. (2007). Quantification of biofilm in microtiter plates: Overview of testing conditions and practical recommendations for assessment of biofilm production by staphylococci. APMIS.

[62] Hennequin C., Forestier C. (2007). Influence of capsule and extended-spectrum beta-lactamases encoding plasmids upon Klebsiella pneumoniae adhesion. Res. Microbiol..

