# Supplementary material for: Phenotypic and Genotypic Features of Klebsiella pneumoniae Harboring Carbapenemases in Egypt: OXA-48-Like Carbapenemases as an Investigated Model
Source: Antibiotics (Basel). 2020 Nov 28;9(12):852. doi: 10.3390/antibiotics9120852 (PMC7760040; doi:10.3390/antibiotics9120852)
Supplement: Supplementary file 1 [file antibiotics-09-00852-s001.pdf]

antibiotics

MDPI

Table S1: Antimicrobial susceptibility patterns of *Klebsiella pneumoniae* isolates by disc diffusion method.

Isolate	Year	Type	Antimicrobial agents																
			AUG 20/10 µg.	TZP 100/10 µg	FOX 30 µg	CTX 30 µg	CTC 30/10 µg	CAZ 30 µg	CZC 30/10 µg	CPM 30 µg	ATM 30 µg	IMI 10 µg	MEM 10 µg	ETR 10 µg	GN 10 µg	AK 30 µg	CIP 5 µg	STX 1.25/ 23.75 µg	TGC 15 µg
1	2014	Wound	R	R	R	R	R	R	R	R	R	R	R	R	R	R	R	R	I
2	2014	Blood	R	R	R	R	R	R	R	R	R	R	R	R	R	R	I	R	R
6	2014	Wound	R	R	R	R	R	R	R	R	R	R	R	R	R	R	I	R	S
7	2014	Urine	R	R	R	R	R	R	R	R	R	R	R	R	S	R	S	S	I
8	2014	Sputum	R	R	R	S	R	R	R	R	R	R	R	R	S	R	I	R	S
14	2014	CVP tip	R	R	R	R	R	R	R	R	R	R	R	R	R	R	I	R	I
16	2014	Wound	R	R	R	R	R	R	R	R	R	R	R	R	S	R	S	S	S
21	2014	Urine	R	R	R	R	R	R	R	R	R	R	R	R	R	R	R	R	S
24	2014	Urine	R	R	R	R	R	R	R	R	R	R	R	R	R	R	R	R	I
28	2014	Sputum	R	R	R	R	R	R	R	R	S	R	R	R	R	S	R	R	I
31	2014	Urine	R	R	R	R	R	R	R	R	R	R	R	R	R	R	R	R	I
33	2014	Urine	R	R	R	R	R	R	R	I	R	I	R	R	R	R	R	R	I
97	2015	Urine	R	R	R	R	R	R	R	R	R	R	R	R	R	R	R	R	R
100	2015	CSF	R	R	R	R	R	R	R	R	R	R	R	R	R	R	R	R	I
102	2015	Blood	R	R	R	R	R	R	R	R	R	R	R	R	R	S	R	R	I
109	2015	Urine	R	R	R	R	R	R	R	R	R	R	R	R	R	R	R	R	I
110	2015	Sputum	R	R	R	R	R	R	R	R	R	R	R	R	R	R	I	R	I
112	2015	Blood	R	R	R	R	R	R	R	R	R	R	R	R	R	R	R	R	R
113	2015	Blood	R	R	R	R	R	R	R	R	R	R	R	R	R	S	R	R	I
117	2015	Urine	R	R	R	R	R	R	R	R	R	R	R	R	R	R	R	R	I
122	2015	Urine	R	R	R	R	R	R	R	R	I	R	R	R	R	R	R	R	I
125	2015	Wound	R	R	R	R	R	R	R	R	R	R	R	R	R	R	I	R	S
127	2015	Blood	R	R	R	R	R	I	R	R	R	R	R	R	R	R	R	R	I

130	2015	Sputum	R	R	R	R	R	R	R	R	R	R	R	R	R	R	R	R	I
134	2015	Blood	R	R	R	R	R	R	R	R	R	R	R	R	R	R	R	R	I
136	2015	Wound	R	R	R	R	R	R	R	I	R	R	R	R	R	R	I	R	I
137	2015	Sputum	R	R	R	R	R	R	R	R	R	R	R	R	R	R	R	R	R
138	2015	Pleural Fluid	R	R	R	R	R	R	R	R	R	R	R	R	R	R	R	R	R
139	2015	Urine	R	R	R	R	R	R	R	R	R	I	R	R	R	R	I	R	R
142	2015	Urine	R	R	R	R	R	R	R	R	R	R	R	R	R	R	R	R	R
143	2015	Blood	R	R	R	R	R	R	R	R	R	R	R	R	R	R	R	R	R
144	2015	Sputum	R	R	R	R	R	R	R	R	R	R	R	R	R	R	R	R	I
145	2015	Wound	R	R	R	R	R	R	R	R	R	R	R	R	R	R	I	R	S
146	2015	Sputum	R	R	R	R	R	R	R	R	R	R	R	R	R	R	I	R	S
147	2015	Blood	R	R	R	R	R	R	R	R	R	R	R	R	R	R	R	R	R
151	2015	Urine	R	R	R	R	R	R	R	R	R	R	R	R	R	R	R	R	R
152	2015	Urine	R	R	R	R	R	R	R	R	R	R	R	R	R	R	R	R	I
300	2016	Wound	R	R	R	R	R	R	R	R	R	R	R	R	R	R	R	R	I
301	2016	Urine	R	R	R	R	R	R	R	R	R	R	R	R	R	R	R	R	S
302	2016	Urine	R	R	R	R	R	R	R	R	R	R	R	R	S	S	R	R	I
303	2016	Sputum	R	R	R	R	R	R	R	R	R	R	R	R	R	R	S	R	I
304	2016	Sputum	R	R	R	R	R	R	R	R	R	R	R	R	R	R	R	R	I
305	2016	Urine	R	R	R	R	R	R	R	R	R	R	R	R	R	R	R	R	R
306	2016	Wound	R	R	R	R	R	R	R	R	R	R	R	R	R	R	R	R	I
307	2016	Wound	R	R	R	R	R	R	R	R	R	R	R	R	R	R	R	R	S
308	2016	Sputum	R	R	R	R	R	R	R	R	R	R	R	R	R	R	I	R	I
310	2016	Wound	R	R	R	R	R	R	R	R	R	R	R	R	R	R	R	R	I
311	2016	Sputum	R	R	R	R	R	R	R	R	R	R	R	R	I	R	R	R	I
312	2016	Blood	R	R	R	R	R	R	R	R	R	R	R	R	R	I	I	R	R
318	2016	Wound	R	R	R	R	R	R	R	R	R	R	R	R	R	R	R	R	I
319	2016	Blood	R	R	R	R	R	R	R	R	R	R	R	R	R	R	I	R	S
320	2016	Sputum	R	R	R	R	R	R	R	R	R	R	R	R	R	R	R	R	I
321	2016	Sputum	R	R	R	R	R	R	R	R	R	R	R	R	R	R	R	R	S
323	2016	ETA	R	R	R	R	R	R	R	R	R	R	R	R	R	R	R	R	I
324	2016	Urine	R	R	R	R	R	R	R	R	R	R	R	R	R	R	R	R	I

325	2016	Wound	R	R	R	R	R	R	R	R	R	R	R	R	R	R	R	R	I
326	2016	Wound	R	R	R	R	R	R	R	R	R	R	R	R	R	R	R	R	I
327	2016	Urine	R	R	R	R	R	R	R	R	R	R	R	R	R	R	R	R	I
329	2016	Sputum	R	R	R	R	R	R	R	R	R	R	R	R	R	R	R	R	S
333	2016	Wound	R	R	R	R	R	R	R	R	R	R	R	R	S	R	R	R	S
334	2016	Blood	R	R	R	R	R	R	R	R	R	R	R	R	R	R	R	R	I
335	2016	Sputum	R	R	R	R	R	R	R	R	R	R	R	R	R	S	R	R	S
337	2016	Sputum	R	R	R	R	R	R	R	R	R	R	R	R	R	R	R	R	S
344	2016	CVP	R	R	R	R	R	R	R	R	R	R	R	R	R	R	R	R	I
345	2016	ETA	R	R	R	R	R	R	R	R	R	R	R	R	R	R	R	R	I
346	2016	Drain Culture	R	R	R	R	R	R	R	R	R	R	R	R	R	R	R	R	I
347	2016	Urine	R	R	R	R	R	R	R	R	R	R	R	R	S	I	R	S	S
348	2016	Sputum	R	R	R	R	R	R	R	R	R	R	R	R	R	R	R	R	I
349	2016	Sputum	R	R	R	R	R	R	R	R	R	R	R	R	R	R	R	R	S
354	2016	Wound	R	R	R	R	R	R	R	R	R	R	R	R	R	R	R	R	I
355	2016	ETA	R	R	R	R	R	R	R	R	R	R	R	R	S	R	R	R	I
356	2016	Wound	R	R	R	R	R	R	R	R	R	R	R	R	R	R	R	R	I
358	2016	Urine	R	R	R	R	R	R	R	R	R	R	R	R	S	S	R	R	I
361	2016	CVL culture	R	R	R	R	R	R	R	R	R	R	R	R	R	R	R	S	S
362	2016	ETA	R	R	R	R	R	R	R	R	R	R	R	R	R	I	R	S	I
365	2016	Urine	R	R	R	R	R	R	R	R	R	R	R	R	R	R	R	R	I
366	2016	Wound	R	R	R	R	R	R	R	R	R	R	R	R	S	R	R	R	I
367	2016	Sputum	R	R	R	R	R	R	R	R	R	R	R	R	R	R	R	R	S
368	2016	Urine	R	R	R	R	R	R	R	R	R	R	R	R	R	R	R	R	I
369	2016	Wound	R	R	R	R	R	R	R	R	R	I	R	R	R	R	R	R	I
371	2016	Sputum	R	R	R	R	R	R	R	R	R	R	R	R	I	R	R	R	S
372	2016	Blood	R	R	R	R	R	R	R	R	R	R	R	R	R	R	R	R	I
373	2016	Sputum	R	R	R	R	R	R	R	R	R	R	R	R	R	R	R	R	S
374	2016	Urine	R	R	R	R	R	R	R	R	R	I	R	R	R	R	R	R	I
375	2016	Urine	R	R	R	R	R	R	R	R	R	R	R	R	R	R	R	R	I
376	2016	Urine	R	R	R	R	R	R	R	R	R	I	R	R	I	R	R	R	I

377	2016	Sputum	R	R	R	R	R	R	R	R	R	R	R	R	I	R	R	R	I
378	2016	Urine	R	R	R	R	R	R	R	R	R	I	R	R	R	R	R	S	I
379	2016	Sputum	R	R	R	R	R	R	R	R	R	I	R	R	I	R	R	S	R
380	2016	Aspirate Fluid	R	I	R	R	R	R	R	I	R	I	R	R	R	R	R	R	I
381	2016	Sputum	R	R	R	R	R	R	R	R	R	R	R	R	R	R	R	R	S
383	2016	ETA	R	R	R	R	R	R	R	R	R	R	R	R	S	R	R	R	S
385	2016	Bile culture	R	R	R	R	R	R	R	R	R	R	R	R	R	R	R	R	I
386	2016	Sputum	R	R	R	R	R	R	R	R	R	I	R	R	R	R	R	R	S
387	2016	Urine	R	R	R	R	R	R	R	R	R	R	R	R	S	R	R	R	R
388	2016	Sputum	R	R	R	R	R	R	R	R	R	R	R	R	R	R	R	R	I
389	2016	Wound	R	R	R	R	R	R	R	R	R	I	R	R	R	R	I	R	I
391	2016	Bed sore	R	R	R	R	R	R	R	R	R	I	I	R	R	R	R	R	I
394	2016	Urine	R	R	R	R	R	R	R	R	R	R	R	R	R	R	R	R	I
396	2016	Wound	R	R	R	R	R	I	R	R	R	R	R	R	R	R	R	R	I
397	2016	Urine	R	R	R	R	R	R	R	R	R	R	R	R	R	R	R	R	I
398	2016	Wound	R	R	R	R	R	R	R	R	R	R	R	R	S	I	R	R	I
399	2016	Wound	R	R	R	R	R	R	R	R	R	R	R	R	R	R	R	R	I
401	2016	Urine	R	R	R	R	R	R	R	R	R	R	R	R	R	R	R	S	I
402	2016	Urine	R	R	R	R	R	R	R	R	R	R	R	R	R	R	R	R	I
403	2016	Urine	R	R	R	R	R	R	R	R	R	R	R	R	R	R	R	S	R
405	2016	Sputum	R	R	R	R	R	R	R	R	R	R	R	R	R	R	R	R	I
407	2016	Urine	R	R	R	R	R	R	R	R	R	R	R	R	I	R	R	R	S
409	2016	Urine	R	R	R	R	R	R	R	R	R	R	R	R	R	R	R	R	R
410	2016	Blood	R	R	R	R	R	R	R	R	R	R	R	R	R	R	R	R	I
411	2016	Wound	R	R	R	R	R	R	R	R	R	R	R	R	R	R	R	R	S
415	2016	Blood	R	R	R	R	R	R	R	R	R	R	R	R	R	R	R	R	I
416	2016	Sputum	R	R	R	R	R	R	R	R	R	R	R	R	R	R	R	R	R
417	2016	Sputum	R	R	R	R	R	R	R	R	R	R	R	R	R	R	R	R	I
418	2016	Urine	R	R	R	R	R	R	R	R	R	R	R	R	R	R	R	R	I
419	2016	Sputum	R	R	R	R	R	R	R	R	R	R	R	R	R	R	R	R	S
420	2016	Blood	R	R	R	R	R	R	R	R	R	R	R	R	R	R	R	I	R

*(AUG) amoxicillin/clavulanic acid, (TZP) piperacillin/tazobactam, (FOX) cefoxitin, (CTX) cefotaxime, (CTC) cefotaxime/clavulanic acid, (CAZ) ceftazidime, (CZC) ceftazidime/clavulanic acid, (CPM) cefepime, (ATM) aztreonam, (IMI) imipenem, (MEM) meropenem, (ETR) ertapenem, (GN) gentamicin , (AK) amikacin, (CIP) ciprofloxacin, (STX) sulfamethoxazole/trimethoprim and (TGC) tigecycline.

R: resistant pattern, I: intermediate pattern, and S: sensitive pattern.

Table S2: Results of modified Hodge test, inhibitor-based tests and genotypic detection of investigated carbapenemases.

Isolate	Phenotypic detection of carbapenemases						Genotypic detection of carbapenemases				
	MHT	Inhibitor based test					Class A	Class B			Class D
		A	B	C	D	TEM(mm)	KPC	NDM	VIM	IMP	OXA-48
1	-	Resistance to meropenem for further comparison with other discs	+	-	-	11	-	+	+	-	-
2	-		+	-	-	12	-	+	+	+	-
6	-		+	-	-	11	-	+	+	+	-
7	-		+	-	-	6	-	+	+	+	-
8	+		-	-	-	6	-	-	-	-	+*
14	-		+	-	-	6	-	+	+	-	-
16	-		+	-	-	10	-	+	-	-	-
21	-		+	-	-	8	-	-	+	+	-
24	-		+	-	-	10	-	-	+	+	-
28	+		-	-	-	6	-	-	-	-	+*
31	-		+	-	-	11	-	-	+	+	-
33	-		+	-	-	13	-	-	+	-	-

97	+		+	-	-	6	-	+	+	+	-
100	+		+	-	-	6	-	+	+	-	-
102	-		-	-	-	6	-	-	+	-	+*
109	-		+	-	-	10	-	-	+	+	-
110	-		+	-	-	11	-	+	+	-	-
112	+		+	-	-	6	-	+	+	+	-
113	+		-	-	-	6	-	-	-	-	+*
117	+		+	-	-	6	-	+	+	+	+#
122	+		+	-	-	12	-	+	+	+	-
125	-		+	-	-	10	-	+	+	+	-
127	+		-	-	-	6	-	-	-	-	+*
130	+		+	-	-	6	-	+	+	+	-
134	+		-	-	-	6	-	+	+	-	+*
136	+		-	-	-	6	-	-	+	-	-
137	+		-	-	-	6	-	-	-	-	-
138	-		+	-	-	10	-	+	+	+	-
139	-		+	-	-	17	-	+	+	+	-
142	+		-	-	-	6	-	-	-	-	-
143	+		+	-	-	14	-	+	-	+	-
144	-		+	-	-	14	-	+	+	-	-
145	-		+	-	-	13	-	+	+	-	-
146	-		+	-	-	12	-	+	+	-	-
147	+		-	-	-	6	-	-	+	-	-
151	+		-	-	-	6	-	-	-	-	+*
152	+		-	-	-	6	-	-	+	-	+*
300	-		+	-	-	15	-	+	+	+	-
301	+		+	-	-	12	-	+	+	+	-
302	+		+	-	-	6	-	+	+	-	-
303	-		-	-	-	6	-	-	+	-	+*
304	+		+	-	-	6	+	+	+	+	-

antibiotics

305	-		+	-	-	10	-	+	+	+	-
306	-		+	-	-	6	-	+	+	+	-
307	-		+	-	-	10	-	+	+	+	-
308	-		+	-	-	11	-	+	+	+	-
310	-		+	-	-	11	-	+	+	+	-
311	+		-	-	-	10	-	-	+	-	+*
312	-		+	-	-	8	-	+	+	+	-
318	-		+	-	-	6	-	+	+	+	-
319	-		+	-	-	9	-	+	+	+	-
320	-		+	-	-	11	-	+	+	+	-
321	+		+	-	-	6	-	+	+	+	-
323	+		+	-	-	6	-	+	+	+	-
324	+		-	-	-	6	-	+	+	-	+*
325	+		+	-	-	6	-	+	+	+	-
326	+		+	-	-	6	-	+	+	+	-
327	-		+	-	-	6	-	+	+	+	-
329	-		+	-	-	10	-	+	+	+	-
333	+		+	-	-	6	-	+	+	-	+#
334	+		-	-	-	6	-	+	+	-	-
335	+		-	-	-	6	-	-	+	-	+*
337	-		+	-	-	18	-	+	+	+	-
344	+		-	-	-	6	-	+	+	-	-
345	-		+	-	-	14	-	+	+	+	-
346	+		-	-	-	6	-	-	-	-	+*
347	-		+	-	-	14	-	+	+	+	-
348	+		+	-	-	6	-	+	+	+	+#
349	+		+	-	-	6	+	+	+	+	+#
354	+		+	-	-	6	+	+	+	+	+#
355	-		+	-	-	6	-	+	+	+	-
356	+		-	-	-	6	-	-	-	-	+*

358	-		+	-	-	12	-	+	+	+	-
361	+		+	-	-	13	-	+	+	+	-
362	-		+	-	-	18	-	+	+	+	-
365	+		-	-	-	6	-	+	+	-	-
366	-		+	-	-	6	-	+	+	+	-
367	+		+	-	-	6	-	+	+	+	-
368	-		+	-	-	9	-	+	+	+	-
369	-		+	-	-	11	-	+	+	+	-
371	+		-	-	-	6	-	+	+	+	+*
372	+		-	-	-	6	-	+	+	+	+*
373	+		-	-	-	6	-	-	+	-	+*
374	-		+	-	-	13	-	-	+	+	-
375	+		-	-	-	6	+	+	+	+	+*
376	-		+	-	-	13	-	+	+	+	-
377	+		-	-	-	6	-	+	+	+	+*
378	+		-	-	-	9	-	-	-	-	+*
379	-		+	-	-	10	-	+	+	+	-
380	-		+	-	-	16	-	+	+	+	-
381	-		+	-	-	13	-	+	+	+	-
383	+		+	-	-	6	-	+	+	+	+#
385	+		-	-	-	6	-	+	+	-	+*
386	-		+	-	-	16	-	+	+	+	-
387	+		-	-	-	6	+	+	+	-	+*
388	+		-	-	-	6	-	+	+	-	-
389	-		+	-	-	11	-	+	+	+	-
391	+		+	-	-	11	-	+	-	+	-
394	+		+	-	-	6	-	+	-	+	+#
396	+		-	-	-	6	-	-	+	-	-
397	+		-	-	-	6	-	+	+	-	+*
398	+		+	-	-	6	-	+	+	-	-

399	+		-	-	-	6	-	-	-	-	+*
401	-		+	-	-	6	-	+	+	+	-
402	+		+	-	-	6	+	+	+	+	-
403	+		+	-	-	6	-	+	+	-	-
405	+		+	-	-	6	-	+	+	+	+#
407	+		+	-	-	8	-	+	+	-	-
409	+		-	-	-	6	-	+	+	-	+*
410	+		-	-	-	6	-	-	-	-	-
411	+		+	-	-	6	-	+	+	+	-
415	+		-	-	-	8	-	-	+	-	+*
416	+		+	-	-	8	-	+	+	+	-
417	+		-	-	-	6	-	+	-	-	-
418	-		+	-	-	6	-	+	+	+	-
419	+		-	-	-	6	-	-	-	-	-
420	-		+	-	-	8	-	+	+	+	-
Disc A	Meropenem 10 μg										
Disc B	Meropenem +EDTA										
Disc C	Meropenem + Phenyl boronic acid										
Disc D	Meropenem +AmpC inhibitor										
TEM	Temocillin 30 μg										
*	Results that do not comply with MβL, KPC and AmpC phenotypic detection criteria and positive genotypically for OXA-48										
#	Results that comply with MβL, KPC or AmpC phenotypic detection criteria and positive genotypically for OXA-48.										

Table S3: Simpson's Diversity Index calculations for antibiogram.

Species Type*	Antibiogram pattern			Isolates	No of individuals (n)	(n-1)	n(n-1)
	S	I	R				
AA	-	-	All investigated antimicrobials	151 & 409	2	1	2
AB	-	TGC	Remaining antimicrobials	117, 134, 152, 324, 346, 348, 354, 356, 372, 375, 385, 394, 397, 399, 405, 415.	16	15	240
AC	-	CAZ& TGC	Remaining antimicrobials	127	1	0	0
AD	-	GN& TGC	Remaining antimicrobials	311& 377	2	1	2
AE	GN& TGC	-	Remaining antimicrobials	333& 383	2	1	2
AF	TGC	-	Remaining antimicrobials	349& 373	2	1	2
AG	GN	-	Remaining antimicrobials	387	1	0	0
AH	TGC	GN	Remaining antimicrobials	371	1	0	0
AI	GN& TGC	CIP	Remaining antimicrobials	8	1	0	0
AJ	CIP	TGC	Remaining antimicrobials	303	1	0	0
AK	AK	TGC	Remaining antimicrobials	102& 113	2	1	2
AL	ATM& AK	TGC	Remaining antimicrobials	28	1	0	0
AM	AK& TGC	-	Remaining antimicrobials	335	1	0	0
AN	STX	IMI& TGC	Remaining antimicrobials	378	1	0	0
							∑ 250

* Species Type:

A: Antibiogram typing method.
(A-N): The patterns of the isolates.

Table S4: Simpson's Diversity Index calculations for ERIC-PCR.

Species Type*	No of individuals(n)	(n-1)	n(n-1)
EA (Isolates 8, 324, 354& 383)	4	3	12
EB (Isolates 117& 397)	2	1	2
EC (Isolate 335)	1	0	0
ED (Isolate 346)	1	0	0
EE (Isolates 385& 387)	2	1	2
EF (Isolates 134, 151, 303, 311, 348, 373, 399, 405, 409& 415)	10	9	90
EG (Isolates 378)	1	0	0
EH (Isolates 28& 356)	2	1	2
EI (Isolates 113)	1	0	0
EJ (Isolates 394)	1	0	0
EK (Isolates 102, 127, 152& 349)	4	3	12
EL (Isolate 333)	1	0	0
EM (Isolate 377)	1	0	0
EN (Isolate 371)	1	0	0
EO (Isolates 372)	1	0	0
EP (Isolates 375)	1	0	0
			∑ 120

* Species Type:
E: ERIC-PCR typing method.
(A-P): The patterns of the isolates.

antibiotics

Table S5: Simpson's Diversity Index calculations for plasmid profile analysis.

Species Type*	No of individuals (n)	(n-1)	n(n-1)
PA1	1	0	0
PA2	1	0	0
PB1	3	2	6
PB2	1	0	0
PC1	1	0	0
PC2	2	1	2
PC3	1	0	0
PC4	1	0	0
PC5	1	0	0
PC6	3	2	6
PC7	1	0	0
PC8	1	0	0
PD1	1	0	0
PD2	1	0	0
PD3	1	0	0
PD4	2	1	2
PD5	1	0	0
PE1	1	0	0
PE2	1	0	0
PE3	1	0	0
PE4	2	1	2
PE5	1	0	0
PE6	1	0	0
PE7	1	0	0
PF1	1	0	0
PG1	1	0	0
			$\sum$ 18

*Species Type:

P: The plasmid profile analysis typing method.

(A-G): The patterns of the isolates, since:

Pattern A has one plasmid.
Pattern B has two plasmids.
Pattern C has three plasmids.
Pattern D has four plasmids.
Pattern E has five plasmids.
Pattern F has six plasmids.
Pattern G has eight plasmids.

antibiotics

MDPI

Table S6: A comparative summing-up of SDI calculations of the three typing methods.

Isolate	Isolate Data		Isolate typing classification		
	Year	Type	Antibiogram	ERIC-PCR	Plasmid Profile analysis
8	2014	Sputum	AI	EA	PC1
28	2014	Sputum	AL	EH	PD1
102	2015	Blood	AK	EK	PE1
113	2015	Blood	AK	EI	PC2
117	2015	Urine	AB	EB	PD2
127	2015	Blood	AC	EK	PB1
134	2015	Blood	AB	EF	PC3
151	2015	Urine	AA	EF	PB1
152	2015	Urine	AB	EK	PC4
303	2016	Sputum	AJ	EF	PA2
311	2016	Sputum	AD	EF	PC6
324	2016	Urine	AB	EA	PC5
333	2016	Wound	AD	EL	PE2
335	2016	Sputum	AM	EC	PC6
346	2016	Drain Culture	AB	ED	PE6
348	2016	Sputum	AB	EF	PE3
349	2016	Sputum	AF	EK	PE4
354	2016	Wound	AB	EA	PE4
356	2016	Wound	AB	EH	PA1
371	2016	Sputum	AH	EN	PG1
372	2016	Blood	AB	EO	PE5
373	2016	Sputum	AF	EF	PF1
375	2016	Urine	AB	EP	PC6
377	2016	Sputum	AD	EM	PE6
378	2016	Urine	AN	EG	PB2
383	2016	ETA	AD	EA	PC7
385	2016	Bile culture	AB	EE	PD4
387	2016	Urine	AG	EE	PC2
394	2016	Urine	AB	EJ	PE7
397	2016	Urine	AB	EB	PD3
399	2016	Wound	AB	EF	PB1
405	2016	Sputum	AB	EF	PC8
409	2016	Urine	AA	EF	PD4
415	2016	Blood	AB	EF	PD5
**Simpson's Diversity Index**			0.78	0.89	0.98

Table S7: Distribution of *fimH-1* and *mrkD* adhesion genes among the carbapenem-resistant isolates.

Isolate	*fimH-1*	*mrkD*	Isolate	*fimH-1*	*mrkD*	Isolate	*fimH-1*	*mrkD*
1	-	+	302	+	+	368	+	+
2	-	+	303	+	+	369	+	+
6	-	+	304	+	+	371	+	+
7	+	+	305	+	+	372	+	+
8	+	+	306	+	+	373	+	+
14	+	+	307	+	+	374	+	+
16	+	+	308	+	+	375	+	+
21	+	+	310	+	+	376	+	+
24	+	+	311	+	+	377	+	+
28	+	+	312	+	+	378	+	+
31	+	+	318	+	+	379	+	+
33	+	+	319	+	+	380	+	+
97	+	+	320	+	+	381	+	+
100	+	+	321	+	+	383	+	+
102	+	+	323	+	+	385	+	+
109	+	+	324	+	+	386	+	+
110	+	+	325	+	-	387	+	+
112	+	+	326	+	+	388	+	+
113	+	+	327	+	+	389	+	+
117	+	+	329	+	+	391	+	+
122	+	+	333	-	+	394	+	+
125	+	+	334	+	+	396	+	+
127	+	+	335	+	+	397	+	+
130	+	+	337	+	+	398	+	+
134	+	+	344	+	-	399	+	+
136	+	+	345	+	+	401	+	+
137	+	+	346	+	+	402	-	+
138	+	+	347	+	+	403	+	+
139	+	+	348	+	+	405	-	+
142	+	+	349	+	+	407	+	+
143	+	+	354	+	+	409	-	+
144	+	+	355	+	+	410	+	+
145	+	+	356	+	+	411	+	+
146	+	+	358	+	+	415	+	+
147	+	+	361	+	+	416	+	+
151	+	+	362	+	+	417	+	+
152	+	+	365	+	+	418	-	+

300	+	+	366	+	+	419	-	+
301	+	+	367	+	+	420	+	+

antibiotics

Table S8: The applied antimicrobial agents, their classes, and their interpretive categories.

Antimicrobial class		Antimicrobial agents	Symbol	Conc.	Interpretive Categories and Zone Diameter Breakpoints (mm)*A		
					**S**	**I**	**R**
**Penicillin Combination Agents.**		Amoxicillin/Clavulanic acid	AUG	20/10 µg	≥ 18	14-17	≤ 13
		Piperacillin/Tazobactam	TZP	100/10 µg	≥ 21	18- 20	≤ 17
**Cephems**	**2nd Generation Cephalosporin**	Cefoxitin	FOX	30 µg	≥ 18	-	≤ 14
	**3rd Generation cephalosporin**	Cefotaxime	CTX	30 µg	≥ 26	23-25	≤ 22
		Cefotaxime/ Clavulanic acid	CTC	40 µg	≥ 26	23-25	≤ 22
		Ceftazidime	CAZ	30 µg	≥ 21	18-20	≤ 17
		Ceftazidime/Clavulanic acid	CZC	40 µg	≥ 21	18-20	≤ 17
	**4th Generation Cephalosporin**	Cefepime	CPM	30 µg	≥ 25	-	≤ 18
**Carbapenems**		Imipenem	IMI	10 µg	≥ 23	20-22	≤ 19
		Meropenem	MEM	10 µg	≥ 23	20-22	≤ 19
		Ertapenem	ETR	10 µg	≥ 22	19-21	≤ 18
**Monobactams**		Aztreonam	ATM	30 µg	≥ 21	18-20	≤ 17
**Aminoglycosides**		Gentamycin	GM	10 µg	≥ 15	13-14	≤ 12
		Amikacin	AK	30 µg	≥ 17	15-16	≤ 14
**Quinolones**		Ciprofloxacin	CIP	5 µg	≥ 21	16-20	≤ 15
**Folate Pathway Inhibitor.**		Trimethoprim./Sulphamethoxazol	SXT	1.25/ 23.75 µg	≥ 16	11-15	≤ 10
**Protein Synthesis inhibitor**		Tigecycline*B	TGC	15 µg	≥ 19	15-18	≤ 14

*A : The interpretation of test results was according to the Clinical and Laboratory Standards Institute guidelines, CLSI (M100-S26, 2016) and further revised according to the recent edition (M100-S28, 2018).

*B: The interpretative results for tigecycline were according to the FDA criteria (http://www.accessdata.fda.gov/drugsatfda_docs/label/2009/021821s016lbl.pdf).

Table S9: Primers sequences and the expected amplicon sizes of investigated genes.

Gene		Primers Sequence (5'-3')	Expected Amplicon Size (bp)	Reference
KPC		KPC- F: 5' ATGTCACTGTATCGCCGTCT 3'	880 bp	[50]
		KPC- R: 5' TTACTGCCCGTTGACGCCCA 3'		
NDM		NDM-F: 5' GGTTTGGCGATCTGGTTTTC 3'	621 bp	[51]
		NDM-R: 5' CGGAATGGCTCATCACGATC 3'		
VIM		VIM-F: 5' GATGGTGTTTGGTCGCATA 3'	390 bp	
		VIM-R: 5' CGAATGCGCAGCACCAG 3'		
IMP		IMP-F: 5' GGAATAGAGTGGCTTAAYTCTC 3'	232 bp	
		IMP-R: 5' GGTTTAAYAAAACAACCACC 3'		
OXA-48-Like genes		OXA-48-F: 5'TTGGTGGCATCGATTATCGG 3'	743 bp	[52]
		OXA-48-R: 5'GAGCACTTCTTTTGTGATGGC 3'		
OXA-48- Like (including flanking or pre-gene)		Pre- OXA-48- F: 5' TATATTGCATTAAGCAAGGG 3'	848 bp	[53]
		Pre- OXA-48-R: 5' CACACAAATACGCGCTAACC 3'		
ERIC-2		5' AAGTAAGTGACTGGGGTGAGCG 3'	---------	[56]
fimH-1	Virulence Adhesion Genes	*fimH-1*-F5' ATGAACGCCTGGTCCTTTGC 3'	688 bp	[34]
		fimH-1-R5' GCTGAACGCCTATCCCCTGC 3'		
mrkD		*mrkD*-F: 5' CCACCAACTATTCCCTCGAA 3'	240 bp	[62]
		mrkD-R: 5' ATGGAACCCACATCGACATT 3'		
